# Cholesterol, Cognition, and Controversy: A Case of Mood Disturbance on Repatha

**DOI:** 10.7759/cureus.87165

**Published:** 2025-07-02

**Authors:** Matteo Conte, Boran Mao, Sara Tahir, Armando Rodriguez, Sayed T Hussain

**Affiliations:** 1 Medicine and Surgery, University of Padova, Padova, ITA; 2 Radiology and Biomedical Imaging, Yale New Haven Hospital, New Haven, USA; 3 Internal Medicine, University of Central Florida College of Medicine, Orlando, USA; 4 Physiology, University of Florida, Gainesville, USA; 5 Cardiology, Iberoamerican University School of Medicine, Santo Domingo, DOM; 6 Interventional Cardiology, University of Central Florida College of Medicine, Orlando, USA

**Keywords:** adverse event, cardiovascular, evolocumab, mood disorder, pcsk9

## Abstract

We report the case of a 52-year-old woman with no prior psychiatric history who developed severe depressive symptoms shortly after starting evolocumab (Repatha) for lipid management. Her symptoms included persistent low mood and crying spells, which resolved completely after discontinuation of the medication. Her low-density lipoprotein (LDL)-cholesterol levels decreased significantly during this period. She was later switched to bempedoic acid and remained asymptomatic. This clinical course suggests a potential link between intensive LDL reduction and mood disturbances. While psychiatric side effects of proprotein convertase subtilisin/kexin type 9 (PCSK9) inhibitors are rarely reported, this case raises awareness and underscores the need for mental health outcomes integration into future PCSK9 investigations.

## Introduction

Cholesterol is the principal component of myelin, and about 25% of the body’s cholesterol is stored in the brain [1]. Despite the widespread use of cholesterol-lowering medications for cardiovascular protection, the functional effect of cholesterol deprivation in the brain is poorly understood. Existing literature highlights an inverse relationship between serum cholesterol levels and mood disorders [2-5]; nonetheless, heterogeneous study designs and broad clinical settings make the association difficult to establish. Does the rate or the extent of cholesterol reduction influence neuropsychiatric outcomes? Could targeting different proteins lead to distinct results? These questions remain unanswered and warrant further investigation.

Evolocumab, a monoclonal antibody inhibiting proprotein convertase subtilisin/kexin type 9 (PCSK9), is among the most potent lipid-lowering agents available. By inhibiting the degradation of low-density lipoprotein (LDL) receptors from the hepatocyte's surface, evolocumab increases the clearance of LDL-cholesterol (LDL-C) from the blood. Although generally well tolerated, neurocognitive and psychiatric adverse effects have been reported for PCSK9 inhibitors in post-marketing pharmacovigilance databases [6,7]. PCSK9 is also expressed in the brain, where it may play a role in synaptic function, cognition, and neuronal survival [8]. Potential drug-drug interactions, such as with frequently co-prescribed beta-blockers, may further affect neuropsychiatric function, although direct evidence remains limited.

To our knowledge, no prior case reports have directly linked evolocumab to the onset of depressive symptoms. This case provides a novel observation and raises clinical considerations on neuropsychiatric safety for patients who require aggressive lipid-lowering medications.

## Case presentation

A 52-year-old woman with a history of coronary artery disease (CAD), atrial and ventricular arrhythmia, type 1 diabetes mellitus, hypertension, and elevated lipoprotein(a) (Lp(a)) was initiated on Repatha 140 mg every two weeks for lipid optimization. Although not formally approved for Lp(a) reduction, PCSK9 inhibitors remain the only FDA-approved drugs shown to lower Lp(a) [9]. In this case, Repatha was selected to address elevated Lp(a) in the context of secondary prevention, despite already optimized LDL-C levels (<70 mg/dL).

Approximately two weeks after starting the medication, she began to experience a gradual onset of depressive symptoms, including unexplained crying spells and persistent low mood. These symptoms progressively worsened over the following weeks, becoming now socially debilitating but without any identifiable external stressors. After eight to nine weeks of treatment, the symptoms had become concerning enough that she contacted the cardiology office to report her mental health changes. She subsequently discontinued the medication on her own after 12 weeks of treatment. Three weeks after discontinuing the medication, the patient reported a complete resolution of depressive symptoms at a follow-up visit. At that time, her LDL-C had decreased from 61 mg/dL to 19 mg/dL and notable shifts in LDL subfractions were observed, including a decrease in small and medium LDL particles. Of note, Lp(a) decreased from 158 to 126 nmol/L (optimal range <75 nmol/L). A summary of the patient's lipid panel throughout the course of PCSK9 treatment is reported in Table 1.

**Table 1:** Serial advanced lipid panel profiles performed before and after Repatha therapy. *Before starting Repatha; **Three weeks after Repatha discontinuation; #Abnormally high values. LDL-C: low-density lipoprotein-cholesterol.

	Baseline*	Four-months after baseline**	Eight-months after baseline	Ten-months after baseline	Optimal range
Cholesterol, total	152	109	146	146	<200 mg/dl
LDL-C	61	19	55	60	<100 mg/dl
LDL particle number	1171#	714	861	1126	<1138 nmol/L
LDL small	208#	108	145	200	<142 nmol/L
LDL medium	205	105	149	160	<215 nmol/L
LDL large	9311	11,477	6133	7354	>6729 nmol/L
LDL peak size	220.5#	223.0	224.2	216.1#	>222.9 Angstrom
Lipoprotein (a)	158#	126#	143#	196#	<75 nmol/L

At the time her depressive symptoms occurred, the patient was taking aspirin 81 mg and rosuvastatin 40 mg daily for cardiovascular protection, metoprolol 25 mg daily for arrhythmia management, subcutaneous insulin (Novolog 100 units/mL) for type 1 diabetes, and amlodipine 5 mg daily for hypertension. This medication regimen had remained stable since April 2020, following an episode of non-ST-elevation myocardial infarction (NSTEMI) treated with a drug-eluting stent in the mid-left anterior descending artery.

The patient actively engages in regular exercise and is a social drinker. She denied tobacco use or illicit drug use. According to her account, there were no notable recent stressors during the period when her depressive symptoms emerged. She has a paternal family history of ischemic heart disease and familial predisposition to blood clots and cancer, but there is no family history of psychiatric conditions.

After Repatha discontinuation, she was switched to bempedoic acid (180 mg daily), with no recurrence of psychiatric symptoms. Subsequent lipid testing performed four and six months after starting bempedoic acid revealed a rebound in small LDL particles and Lp(a). A rechallenge test was not performed due to patient concerns about mood disturbance recurrence, limiting the ability to establish a definitive causal relationship.

This brief report has been prepared following the CARE guidelines to ensure completeness, transparency, and standardized clinical case reporting [10]. Written informed consent was obtained from the patient for the publication of this case report. Per institutional guidelines, IRB approval was not required for a single-patient case report.

The symptom onset following Repatha onset and the subsequent resolution upon discontinuation suggest a potential association between PCSK9 inhibition and mood disorders.

## Discussion

The present case report depicts an early-onset mood disorder attributed to the lipid-lowering medication Repatha in a patient without any history of previous mood disorders. 

Whether lipid-lowering medications could be a cause of mood disorders is debated in the literature. A recurrent trend has been observed between low cholesterol levels and mood disorders, particularly depression and increased suicidality [2-5]. To define the trend, a meta-analysis of 32 cross-sectional studies found that individuals with depression had lower LDL-C values compared to individuals who were screened but did not meet the diagnostic criteria (−4.29 mg/dL, 95% CI: −8.19 to −0.40, p = 0.03) [11]. Although statistically significant, an absolute difference of 4 mg/dl is modest and may hold limited clinical relevance. Low LDL-C was further evaluated as a categorical variable and compared to high LDL-C. The odds of having depression with low LDL-C were not significantly different compared to high LDL-C (OR: 0.90, 95% CI: 0.80-1.01, p = 0.08) [11].

Originally identified as an upregulated gene in cerebellar neurons during apoptosis, the PCSK9 molecule has become one of the most effective targets for cardiovascular protection [12]. Whether the inhibition of PCSK9 could favor the onset of mood disorders was explored by Eudravigilance, the European centralized surveillance database for managing and analyzing reports of suspected adverse reactions to medications [6,7]. Of 258 serious suspected adverse reactions self-reported in Eudravigilance between 2020 and 2023 for evolocumab, eight were psychiatric disorders according to the system organ class (SOC) level [7].

Neuropsychiatric adverse reaction individual reports were then compared between PCSK9 inhibitors and statins. While neurologic adverse events were overall more commonly reported in PCSK9 inhibitors, psychiatric symptoms were reported less often in evolocumab than simvastatin (reported OR: 0.56, 95% CI: 0.49-0.64, p < 0.001) [6]. A similar trend is suggested when evolocumab is compared with high-potency statins (rosuvastatin ROR: 0.68, 95% CI: 0.60-0.78, p <0.001; atorvastatin ROR: 0.96, 95% CI: 0.85-1.09, p = 0.546) [6]. To help contextualize these pharmacovigilance findings, we summarized the rate of neuropsychiatric adverse drug reactions (ADRs) reported for PCSK9 inhibitors and statins in Table 2. While reporting odds ratios (RORs) reflect the strength of a statistical signal across individual case safety reports (ICSRs), ADR counts provide a sense of clinical burden and represent an interpretable metrics for clinicians assessing PCSK9 risk profile. 

**Table 2:** Overview of reported neuropsychiatric ADRs in patients treated with PCSK9 inhibitors and statins in Europe (2015–2020). Each report was frequently filed by healthcare professionals (72.3% of cases) and covered multiple ADRs. ADR: adverse drug reaction; PCSK9: proprotein convertase subtilisin/kexin type 9.

	Evolocumab	Alirocumab	Simvastatin	Atorvastatin
Target population (estimate)	~500,000 patients		~100 million patients	
Nervous system ADRs (n)	1406	975	2035	2450
Psychiatric ADRs (n)	468	321	1131	1085
Insomnia	38	41	267	209
Depression	31	44	136	140
Confusion	28	39	80	85

The relationship between lipid-lowering therapies and mood disorders remains complex, particularly for widely prescribed agents such as statins. A meta-analysis evaluating the adjunctive use of statins in patients with major depressive disorder (MDD) found that, compared to selective serotonin reuptake inhibitors (SSRIs) alone, statins and SSRIs combination was associated with a reduction in the Hamilton depression rating scale (HDRS) scores (mean difference: −2.79; 95%: CI −3.83 to −1.76, p < 0.001) [13].

Brain functions of PCSK9 were recently evaluated in animal studies by creating a knock-out of the culprit protein in mice. A downregulation of PCSK9 expression led to impaired synaptic plasticity of hippocampal dendritic spines, thus affecting cognitive function and performance [8]. Interestingly, patients with MDD display a similar pattern of hippocampal synaptic dysfunction [14]. Although a reduction in dendritic spine length observed in mice differs from a reduction in dendritic spine number reported in human functional imaging studies [8,14], both findings suggest structural alteration of synaptic integrity. Evaluating the long-term results of PCSK9 downregulation in humans appear to be crucial, as accounting for neuropsychiatric side effects can affect cardiovascular management in selected populations.

Lipoprotein(a) inverse trend

While an inverse relationship between LDL-C and depression exists, not all lipid particles appear to behave the same way. Lp(a) is structurally a variant of LDL particle covalently attached to Apo(a) via disulfide bond [15]. The Apo(a) appears to act like a plasminogen, with the potential to cause plaque thrombosis, hereby explaining the link with increased cardiovascular disease [15]. Little is known about the non-cardiovascular effects of high Lp(a). Hamidifard et al. investigated the potential role of Lp(a) in patients with and without depression [4]. The lipid particle level was significantly higher in patients with major depression (34.94 ± 18.01 vs. 20.08 ± 11.27 mg/dl in the controls). The association was significantly different (p < 0.001) between the two groups.

Future directions

While aggressive LDL-C lowering with PCSK9 inhibitors offers undoubted cardiovascular protection, extremely low LDL-C levels may have unintended effects. Understanding the broader systemic roles of lipid particles could help explain rare adverse reactions, including mood disturbances. Clinicians may consider monitoring for neuropsychiatric symptoms in select patients, especially those achieving very low LDL-C levels.

## Conclusions

This case report highlights a potential link between PCSK9 inhibition and mood disturbances, underscoring the need for prospective studies and post-marketing surveillance. The onset and resolution of symptoms in close temporal relation to Repatha therapy raise clinical concern, particularly for individuals undergoing aggressive LDL-C lowering. While causality cannot be established, the observed response underscores the value of neuropsychiatric monitoring during treatment. Further clinical evaluation is needed to clarify whether such effects are isolated or underrecognized, and whether certain patients may be more vulnerable to mood changes in this context.
